# Mineralocorticoid Receptor Blockade Mitigates Loop Diuretic-Exacerbated Plasma Volume Contraction and Target-Organ Fibrosis in Angiotensin II-Induced Hypertension

**DOI:** 10.3390/ijms27188251

**Published:** 2026-09-16

**Authors:** Junxian Liu, Yin Hua Zhang

**Affiliations:** 1 Department of Physiology & Biomedical Sciences, Ischemic/Hypoxic Disease Institute, Seoul National University College of Medicine, Seoul 03080, Republic of Korea; jushin@snu.ac.kr; 2 Yanbian University Hospital, Yanbian University, Yanji 133000, China

**Keywords:** hypertension, furosemide, spironolactone, fibrosis, plasma volume

## Abstract

Diuretics are widely used antihypertensive agents because of their potent natriuretic effects. However, in hypertension with excessive renin–angiotensin–aldosterone system (RAAS) activation, further volume depletion may aggravate target-organ injury. We investigated the effects of loop diuretics and mineralocorticoid receptor (MR) blockade on plasma volume (PV) and organ injury in angiotensin II (Ang II)-induced hypertension. Male Sprague–Dawley rats were assigned to six groups (n = 6/group) receiving Sham or Ang II with or without furosemide and spironolactone. PV was measured by indocyanine green (ICG) dilution, together with blood pressure, histological, immunohistochemical, and molecular assessments. Longitudinal data were analyzed by two-way repeated-measures ANOVA; animal-level endpoints by one-way ANOVA; histological and immunohistochemical measurements by nested one-way ANOVA; and two-group comparisons by unpaired *t* tests, with appropriate post hoc testing. Ang II reduced PV, whereas furosemide caused a further decline without additional blood pressure reduction, accompanied by aggravated fibrosis, endothelial impairment, and dysregulation of fibrosis-related proteins. Spironolactone attenuated PV contraction, lowered blood pressure, preserved endothelial integrity, alleviated fibrosis, and partially normalized molecular alterations. MR blockade was associated with attenuation of PV contraction and target-organ injury, supporting a potential relationship between PV homeostasis and organ protection during chronic loop diuretic treatment.

## 1. Introduction

Hypertension is a major risk factor for cardiovascular and chronic kidney diseases. Persistent activation of the RAAS, particularly overactivation of Ang II signaling, plays a central role in the pathogenesis of hypertension by promoting sustained vasoconstriction, vascular remodeling, inflammation, and fibrosis, ultimately resulting in target organ damage [1,2]. Diuretics are widely recommended as first-line antihypertensive agents because of their ability to reduce sodium retention and extracellular fluid volume [3]. However, plasma volume is not uniformly expanded in hypertension. Classical and contemporary clinical studies have demonstrated substantial heterogeneity in intravascular volume status, with PV being normal or reduced in a subset of hypertensive individuals [4,5,6,7]. Instead, patients with high-renin hypertension and experimental models characterized by excessive Ang II activity often exhibit relative contraction of effective circulating PV despite persistent hypertension [8,9]. This raises the possibility that further plasma volume depletion induced by diuretics, especially loop diuretics, may not always be beneficial under conditions of marked RAAS activation.

Loop diuretics inhibit the Na^+^-K^+^-2Cl^−^ cotransporter (NKCC2) in the thick ascending limb of the loop of Henle, thereby producing potent natriuresis and diuresis with consequent reduction in extracellular fluid and PV [10]. However, excessive volume depletion may trigger compensatory activation of the RAAS and sympathetic nervous system, leading to increased vasoconstriction, renal hypoperfusion, and electrolyte disturbances [11,12]. Under conditions of high blood pressure, in which PV is relatively contracted, these compensatory responses may attenuate the antihypertensive efficacy of loop diuretics and increase susceptibility to cardiovascular and renal injury.

Previous studies have established that loop diuretics reduce intravascular volume and may activate compensatory neurohumoral pathways, whereas MR antagonists exert cardiovascular and renal protective effects beyond blood pressure reduction [13,14,15,16]. However, these effects have largely been investigated separately. In particular, it remains unclear whether chronic loop diuretic-induced PV contraction is associated with aggravated target-organ injury under conditions of excessive RAAS activation, and whether the protective effects of MR blockade during concomitant loop diuretic therapy are accompanied by attenuation of PV contraction. Moreover, few studies have integrated direct longitudinal PV measurements with simultaneous assessment of cardiovascular and renal structural, endothelial, and molecular remodeling in this setting.

Therefore, the present study compared the effects of furosemide monotherapy and combined furosemide plus spironolactone treatment in an Ang II-induced hypertensive rat model. PV was directly quantified using the ICG indicator dilution method, together with longitudinal blood pressure assessment and histological and molecular analyses of the heart, kidney, and aorta. We hypothesized that furosemide-induced further PV contraction would be associated with aggravated target-organ injury, whereas MR blockade would attenuate both PV contraction and pathological remodeling.

## 2. Results

### 2.1. BP and PV Changes with Furosemide and Spironolactone in Ang II-Induced Hypertension

**Figure 1:**
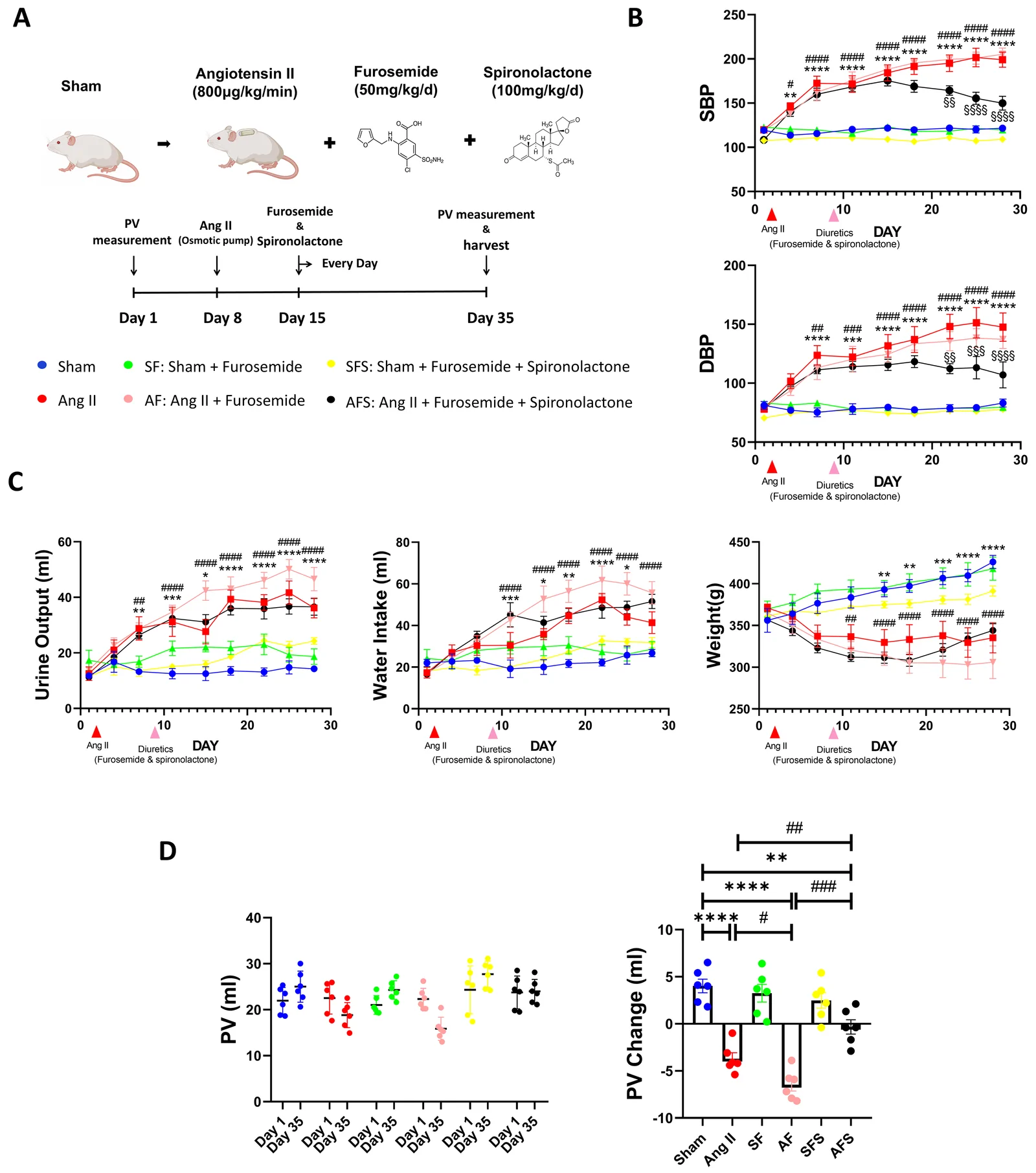
Experimental design, systolic blood pressure (SBP), diastolic blood pressure (DBP), urine output, water intake, body weight, and changes in plasma volume (ΔPV). (**A**) Experimental design. Plasma volume (PV) was measured in all rats on the first experimental day (Day 1). Seven days after PV measurement (Day 8), Ang II was continuously infused (800 ng/kg/min) using an osmotic pump. Seven days after pump implantation (Day 15), oral administration of furosemide (50 mg/kg/day) or furosemide plus spironolactone (100 mg/kg/day) was initiated. On Day 35 (28 days after pump implantation), PV was measured again immediately before euthanasia. Rats were allocated into six groups according to the treatment protocol: Sham, Ang II, SF, AF, SFS, and AFS, with six rats in each group. (**B**) Comparison of SBP and DBP among the experimental groups. Values are expressed as mean ± SD. Sham vs. Ang II, ** *p* < 0.01, *** *p* < 0.001, **** *p* < 0.0001; Sham vs. AF, # *p* < 0.05, ## *p* < 0.01, ### *p* < 0.001, #### *p* < 0.0001; Ang II vs. AFS, §§ *p* < 0.01, §§§ *p* < 0.001, §§§§ *p* < 0.0001; n = 6 per group. (**C**) Comparison of urine output, water intake, and body weight among the experimental groups. Values are expressed as mean ± SD. Sham vs. Ang II, * *p* < 0.05, ** *p* < 0.01, *** *p* < 0.001, **** *p* < 0.0001; Sham vs. AF, ## *p* < 0.01, #### *p* < 0.0001; n = 6 per group. (**D**) Plasma volume (PV) measured at Day 1 and Day 35, and the corresponding changes in PV (ΔPV) in each experimental group. Values are expressed as mean ± SD. Sham vs. Ang II; Sham vs. AF, **** *p* < 0.0001; Sham vs. AFS, ** *p* < 0.01; Ang II vs. AF, # *p* < 0.05; Ang II vs. AFS, ## *p* < 0.01; AF vs. AFS, ### *p* < 0.001; n = 6 per group.

As shown in Figure 1B, systolic blood pressure (SBP) and diastolic blood pressure (DBP) began to increase on the third day after implantation of the Ang II-infusing osmotic pump and remained elevated throughout the experimental period. Peak BP values were as follows: Sham, SBP 114.69 ± 4.80 mmHg and DBP 84.67 ± 5.13 mmHg; Ang II, SBP 208.73 ± 19.72 mmHg and DBP 157.71 ± 27.87 mmHg (Sham vs. Ang II *p* < 0.0001; Figure 1B). One week after osmotic pump implantation, oral furosemide treatment was initiated. No reduction in BP was observed in the AF group, and BP remained unchanged in the SF group. Peak BP values were as follows: SF, SBP 126.34 ± 6.71 mmHg and DBP 87.79 ± 4.71 mmHg; AF, SBP 210.56 ± 11.84 mmHg and DBP 151.83 ± 20.01 mmHg (Sham vs. AF *p* < 0.0001; Figure 1B). In contrast, combined treatment with furosemide and spironolactone significantly reduced BP in the AFS group from Day 14 after treatment initiation onward, whereas BP remained unchanged in the SFS group. The final BP measured before euthanasia was as follows: SFS, SBP 109.00 ± 5.02 mmHg and DBP 77.83 ± 4.86 mmHg; AFS, SBP 149.83 ± 19.55 mmHg and DBP 96.17 ± 6.37 mmHg. At the final measurement, although BP in the AFS group remained above the normal range, both SBP and DBP were significantly lower than those in the Ang II and AF groups (Ang II vs. AFS *p* < 0.0001; AF vs. AFS *p* < 0.0001 Figure 1B). 

In the NHANES cohort, hypertensive patients receiving loop diuretic monotherapy had significantly higher systolic blood pressure (SBP) than those receiving combination therapy with loop diuretics and MR blockade. Although diastolic blood pressure (DBP) also tended to be higher in the loop diuretic monotherapy group, the difference was not statistically significant. Detailed results are provided in Supplementary Table S1A. Multivariable linear regression analysis using the loop diuretic-only group as the reference showed that concomitant MR blockade was associated with significantly lower SBP after adjustment for age, sex, BMI, heart failure, diabetes mellitus, eGFR, RBC, and WBC (B = −12.704 mmHg, 95% CI: −17.447 to −7.962; *p* < 0.001). DBP showed a similar direction of association, although the association did not reach statistical significance (B = −2.764 mmHg, 95% CI: −5.910 to 0.381; *p* = 0.085). Detailed results are provided in Supplementary Table S1B.

Compared with the Sham group, both the Ang II and AF groups exhibited progressive increases in urine output and water intake, accompanied by a reduction in body weight (Figure 1C). In the SF and SFS groups, only modest increases in urine output and water intake were observed. In the AFS group, urine output and water intake remained comparable to those in the Ang II group throughout most of the experimental period (Figure 1C). Ang II infusion also induced progressive body weight loss, which was further exacerbated in the AF group. Although body weight in the AFS group remained lower than that in the Sham group, it showed a noticeable recovery during the late stage of the experiment (Figure 1C).

The baseline PV measured on the first experimental day (Day 1) showed no significant differences among the experimental groups. After 35 days of intervention, PV exhibited distinct changes depending on the treatment conditions (Figure 1D, left). Compared with the Sham group, Ang II infusion significantly reduced PV, and concomitant furosemide administration further decreased PV (Figure 1D, right). In contrast, combined treatment with furosemide and spironolactone significantly attenuated the reduction in PV observed in the Ang II group (Figure 1D, right). PV was increased in the Sham, SF and SFS groups, and the magnitude of PV increase did not differ significantly among the three groups. The mean changes in PV (ΔPV) were as follows: Sham, +4.02 ± 1.79 mL; Ang II, −3.70 ± 1.51 mL; SF, +3.25 ± 2.29 mL; AF, −6.48 ± 1.61 mL; SFS, +2.47 ± 2.03 mL; and AFS, −0.33 ± 1.85 mL (Figure 1D, right). Detailed PV measurement data are provided in Supplementary Table S2.

### 2.2. Protective Effects of Spironolactone Against Ang II- and Furosemide-Induced Histopathology

**Figure 2:**
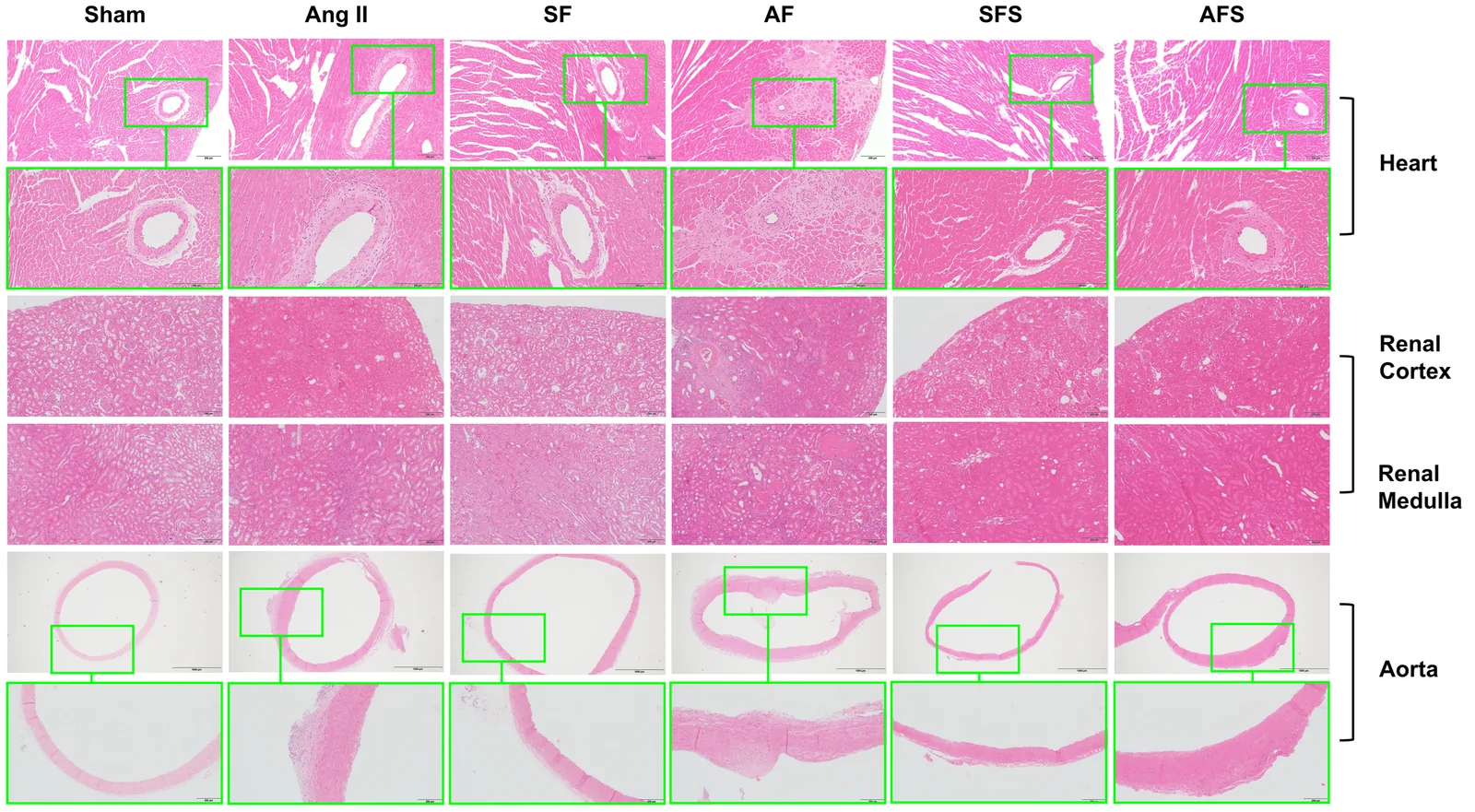
Representative H&E-stained images of the heart, kidney, and aorta. The upper two rows show the heart (upper: ×100; lower: ×200), the middle two rows show the renal cortex and medulla (upper: ×100; lower: ×100), and the lower two rows show the aorta (upper: ×40; lower: ×100).

H&E staining revealed marked histopathological changes in the Ang II group, including extracellular matrix accumulation surrounding intramyocardial vessels and in the adventitia of the aorta (Figure 2, rows 1–2 and 5–6). In the kidney, numerous collapsed and sclerotic glomeruli were observed in the renal cortex, accompanied by tubular atrophy with structural distortion in the renal medulla (Figure 2, rows 3–4). Compared with the Ang II group, the AF group displayed more severe pathological alterations, including pronounced narrowing of the intramyocardial vascular lumen with extensive degeneration of the surrounding myocardium (Figure 2, rows 1–2). The aorta showed focal intimal protrusions, while renal injury was aggravated, characterized by more widespread tubular damage and prominent tubular cast formation (Figure 2, rows 3–6). In the AFS group, only mild extracellular matrix accumulation was observed around cardiac vessels and aortic adventitia, whereas the number of sclerotic glomeruli was substantially reduced and renal tubular architecture was largely preserved (Figure 2). No obvious histopathological abnormalities were observed in the Sham, SF, or SFS groups.

**Figure 3:**
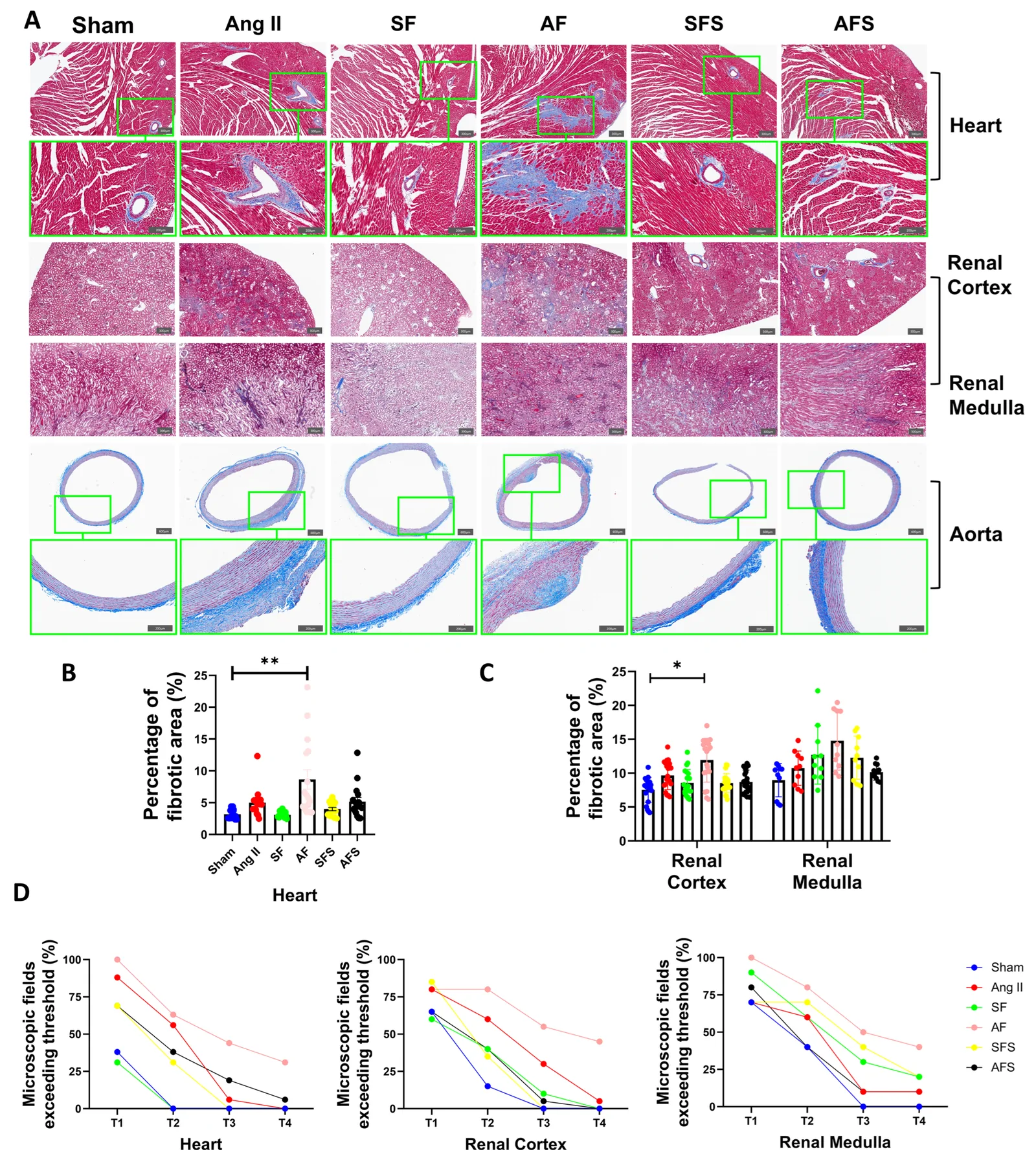
Representative Masson’s trichrome staining images and quantitative analysis of fibrotic area. (**A**) Representative Masson’s trichrome-stained images of the heart, kidney, and aorta. The upper two rows show the heart (upper: ×100; lower: ×200), the middle two rows show the renal cortex and medulla (upper: ×100; lower: ×100), and the lower two rows show the aorta (upper: ×40; lower: ×100). (**B**) Quantitative analysis of the fibrotic area in cardiac microscopic fields acquired at ×200 magnification. A total of 16 microscopic fields per group were obtained from tissue sections of two rats. Each data point represents one microscopic field, and values are shown as mean ± SD. Statistical comparisons were performed using nested one-way ANOVA, with the individual animal as the biological experimental unit and microscopic fields nested within-animal. Sham vs. AF, ** *p* < 0.01. (**C**) Quantitative analysis of the fibrotic area in the renal cortex and medulla using microscopic fields acquired at ×100 magnification. Twenty cortical and ten medullary microscopic fields per group were obtained from tissue sections of two rats. Each data point represents one microscopic field, and values are shown as mean ± SD. Separate nested one-way ANOVAs were performed for the renal cortex and medulla, with the individual animal as the biological experimental unit and microscopic fields nested within-animal. In the renal cortex, Sham vs. AF, * *p* < 0.05. No statistically significant between-group differences were detected in the renal medulla. (**D**) Exploratory threshold-based analysis of the distribution of fibrosis severity in the heart, renal cortex, and renal medulla. T1 was defined as the mean fibrotic area of microscopic fields in the Sham group for each tissue. For the heart, T2, T3, and T4 corresponded to 1.5-, 2.0-, and 3.0-fold T1, respectively; for the renal cortex and medulla, T2, T3, and T4 corresponded to 1.2-, 1.5-, and 1.8-fold T1, respectively. The percentage of microscopic fields exceeding each threshold was calculated for each experimental group. This analysis was performed descriptively, and no inferential statistical comparisons were applied.

Masson’s trichrome staining confirmed that extracellular matrix accumulation observed around intramyocardial vessels and in the aortic adventitia of Ang II-treated rats represented fibrotic lesions (Figure 3A, rows 1–2 and 5–6). In the kidney, marked collagen deposition was also detected within glomeruli and the peritubular interstitium (Figure 3A, rows 3–4). Compared with the Ang II group, the AF group exhibited more extensive fibrosis, with collagen deposition extending from the perivascular regions into the surrounding myocardium in the heart, from the adventitia toward the intima in the aorta, and involving a substantially larger area of the renal cortex and medulla (Figure 3A). In the AFS group, fibrotic changes were markedly alleviated in all examined organs (Figure 3A, rows 1–6). Notably, the number of severely fibrotic glomeruli was lower than that observed in the Ang II group (Figure 3A).

Quantitative analysis of the fibrotic area generally supported the histological findings. In the heart, fibrotic area was significantly greater in the AF group than in the Sham group (Figure 3B). Similarly, renal cortical fibrosis was significantly increased in the AF group compared with the Sham group (Figure 3C). The AF group generally showed higher mean fibrotic area, whereas lower values were observed in the AFS group; however, these differences did not reach statistical significance in the other comparisons. No significant between-group differences were detected in the renal medulla (Figure 3C). Detailed results of the corresponding hierarchical statistical analyses are provided in Supplementary Table S3. Detailed quantitative fibrotic area measurements data are provided in Supplementary Table S4.

To further characterize the distribution of fibrosis severity, an exploratory threshold-based analysis was performed (Figure 3D). As the threshold increased from T1 to T4, the proportion of microscopic fields exceeding the threshold progressively decreased in all groups. Notably, the AF group retained a greater proportion of fields exceeding the higher thresholds (T3 and T4), particularly in the heart and renal cortex, indicating a greater prevalence of fields with relatively severe fibrosis. A similar pattern was observed in the renal medulla. In contrast, the proportion of high-threshold fields was lower in the AFS group than in the AF group. These descriptive findings were consistent with the histological observation of more pronounced and heterogeneous fibrotic involvement in the AF group and its attenuation with concomitant MR blockade.

### 2.3. Spironolactone Preserves Vascular Endothelial Integrity in Ang II- and Furosemide-Treated Rats

**Figure 4:**
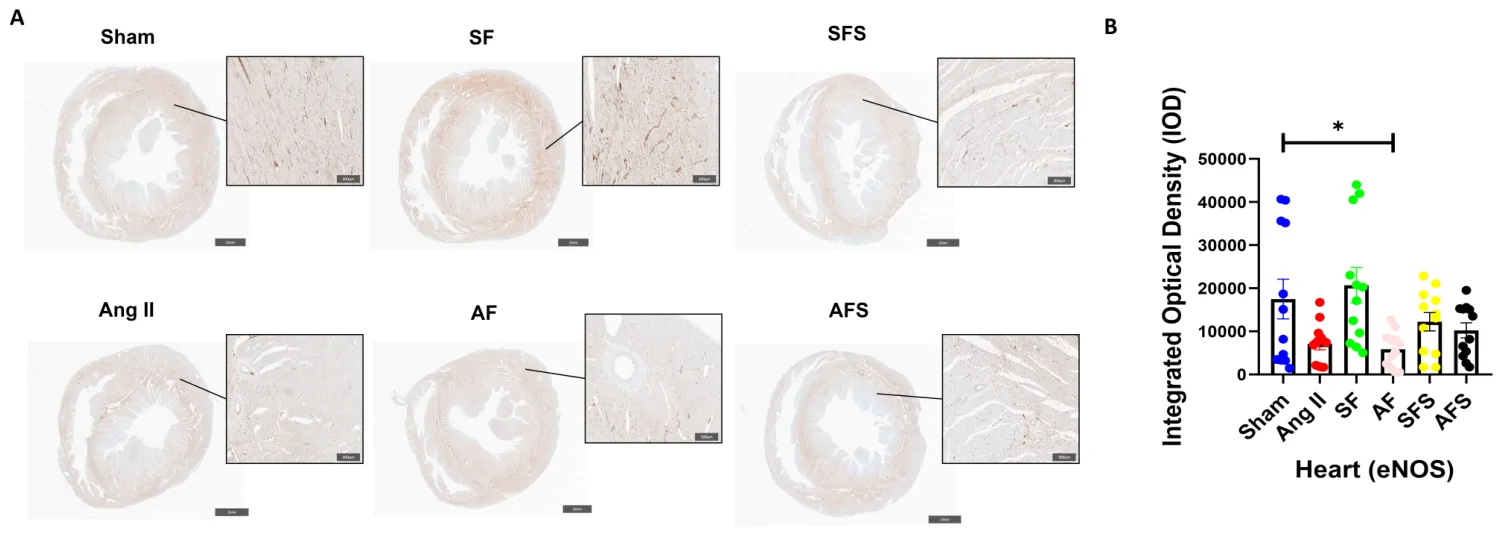
Representative eNOS immunohistochemical images and quantitative analysis of eNOS staining intensity in the heart. (**A**) Representative eNOS IHC images of the heart. Low-magnification images are shown at ×10, and high-magnification images are shown at ×100. (**B**) Quantitative analysis of eNOS staining intensity in the heart. A total of 12 microscopic fields per group were obtained from tissue sections of two rats. Each data point represents one microscopic field, and values are shown as mean ± SD. Statistical comparisons were performed using nested one-way ANOVA, with the individual animal as the biological experimental unit and microscopic fields nested within-animal. Sham vs. AF, * *p* < 0.05.

Because the histological abnormalities identified by H&E and Masson’s trichrome staining were predominantly localized around blood vessels, endothelial integrity was further assessed by eNOS immunohistochemistry (Figure 4A). Quantitative analysis showed that eNOS staining intensity was significantly lower in the AF group than in the Sham group (Figure 4B). The Ang II group also showed a lower mean eNOS staining intensity than the Sham group, whereas a relatively higher value was observed in the AFS group compared with the AF group; however, these differences did not reach statistical significance (Figure 4B).

**Figure 5:**
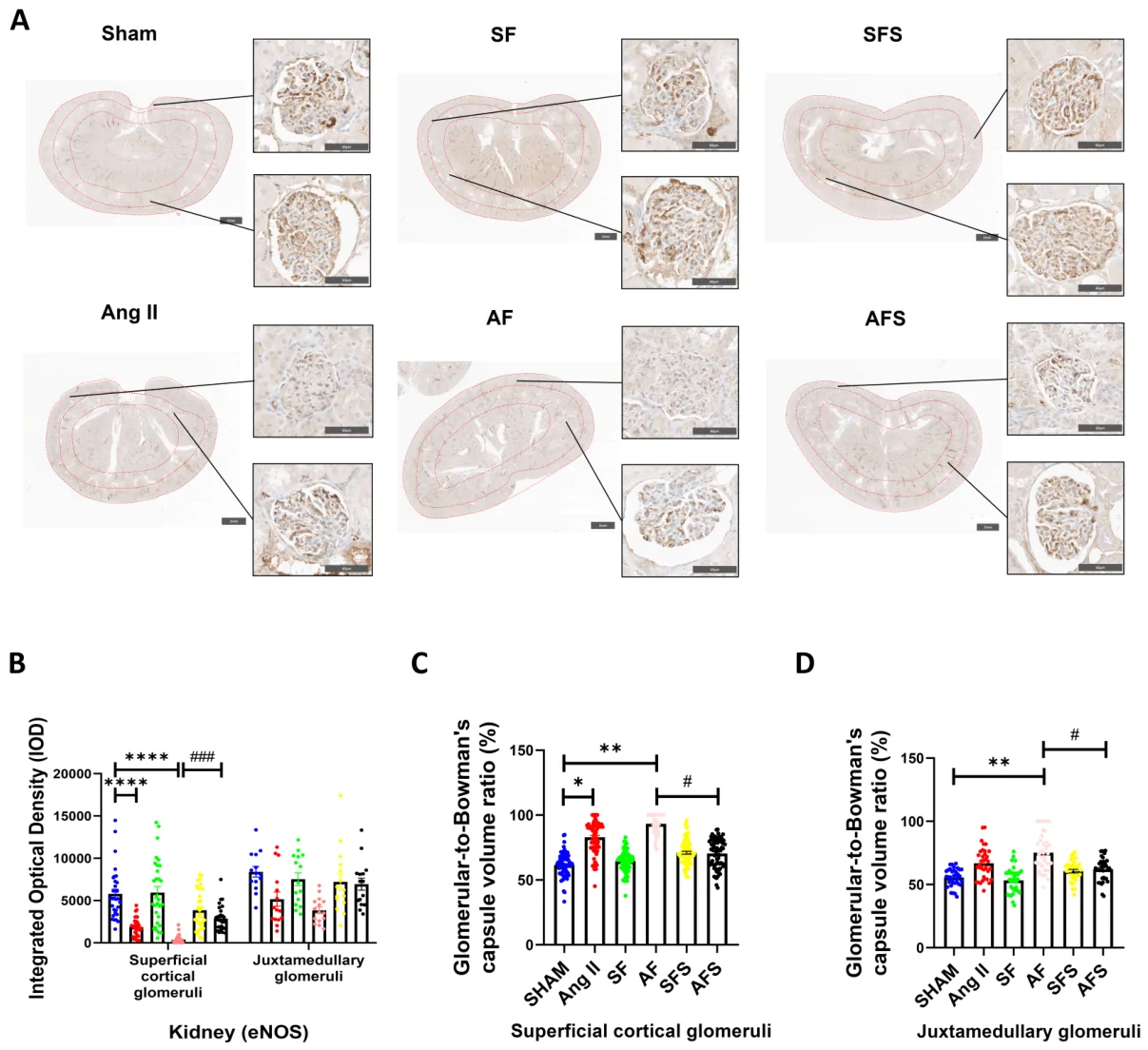
Renal eNOS immunohistochemistry and quantitative assessment of glomerular structural alterations. (**A**) Representative eNOS immunohistochemical images of superficial cortical and juxtamedullary glomeruli. Low-magnification images are shown at ×10, and high-magnification images are shown at ×400. (**B**) Quantitative analysis of eNOS staining intensity in superficial cortical and juxtamedullary glomeruli. Thirty superficial cortical glomeruli and fifteen juxtamedullary glomeruli per group were obtained from tissue sections of two rats. Each data point represents one glomerulus, and values are shown as mean ± SD. Separate nested one-way ANOVAs were performed for superficial cortical and juxtamedullary glomeruli, with the individual animal as the biological experimental unit and glomeruli nested within-animal. **Superficial cortical glomeruli**: Sham vs. Ang II, **** *p* < 0.0001; Sham vs. AF, **** *p* < 0.0001; AF vs. AFS, ### *p* < 0.001. No statistically significant differences were detected among the prespecified comparisons in **juxtamedullary glomeruli**. (**C**) Quantitative analysis of the glomerular-to-Bowman’s capsule ratio in superficial cortical glomeruli. Seventy glomeruli per group were obtained from tissue sections of two rats. Each data point represents one glomerulus, and values are shown as mean ± SD. Statistical comparisons were performed using nested one-way ANOVA, with the individual animal as the biological experimental unit and glomeruli nested within-animal. Sham vs. Ang II, * *p* < 0.05; Sham vs. AF, ** *p* < 0.01; AF vs. AFS, # *p* < 0.05. (**D**) Quantitative analysis of the glomerular-to-Bowman’s capsule ratio in juxtamedullary glomeruli. Thirty-five glomeruli per group were obtained from tissue sections of two rats. Each data point represents one glomerulus, and values are shown as mean ± SD. Statistical comparisons were performed using nested one-way ANOVA, with the individual animal as the biological experimental unit and glomeruli nested within-animal. Sham vs. AF, ** *p* < 0.01; AF vs. AFS, # *p* < 0.05.

In the kidney, eNOS immunoreactivity was evaluated separately in superficial cortical and juxtamedullary glomeruli to assess glomerular endothelial changes at different cortical depths (Figure 5A). In superficial cortical glomeruli, eNOS staining intensity was significantly reduced in both the Ang II and AF groups compared with the Sham group (Figure 5B). In contrast, eNOS staining intensity was significantly higher in the AFS group than in the AF group (Figure 5B). In juxtamedullary glomeruli, although lower mean eNOS staining intensity was observed in the Ang II and AF groups and a relatively higher value was observed in the AFS group, these differences did not reach statistical significance after nested analysis (Figure 5B). Detailed cardiac and renal eNOS staining intensity data are provided in Supplementary Table S5.

To further assess glomerular structural alterations, the glomerular-to-Bowman’s capsule ratio was quantified in superficial cortical and juxtamedullary glomeruli. In superficial cortical glomeruli, the ratio was significantly increased in the Ang II group and AF group compared with the Sham group (Figure 5C). The ratio was significantly lower in the AFS group than in the AF group (Figure 5C). In juxtamedullary glomeruli, the ratio was significantly increased in the AF group compared with the Sham group, whereas the AFS group showed a significantly lower ratio than the AF group (Figure 5D). No statistically significant differences were detected in the other prespecified comparisons. The individual glomerular and Bowman’s capsule measurements used to calculate these ratios are provided as supporting data in Supplementary Figure S1 and Supplementary Table S6.

### 2.4. Expression of Fibrosis-Related Proteins

**Figure 6:**
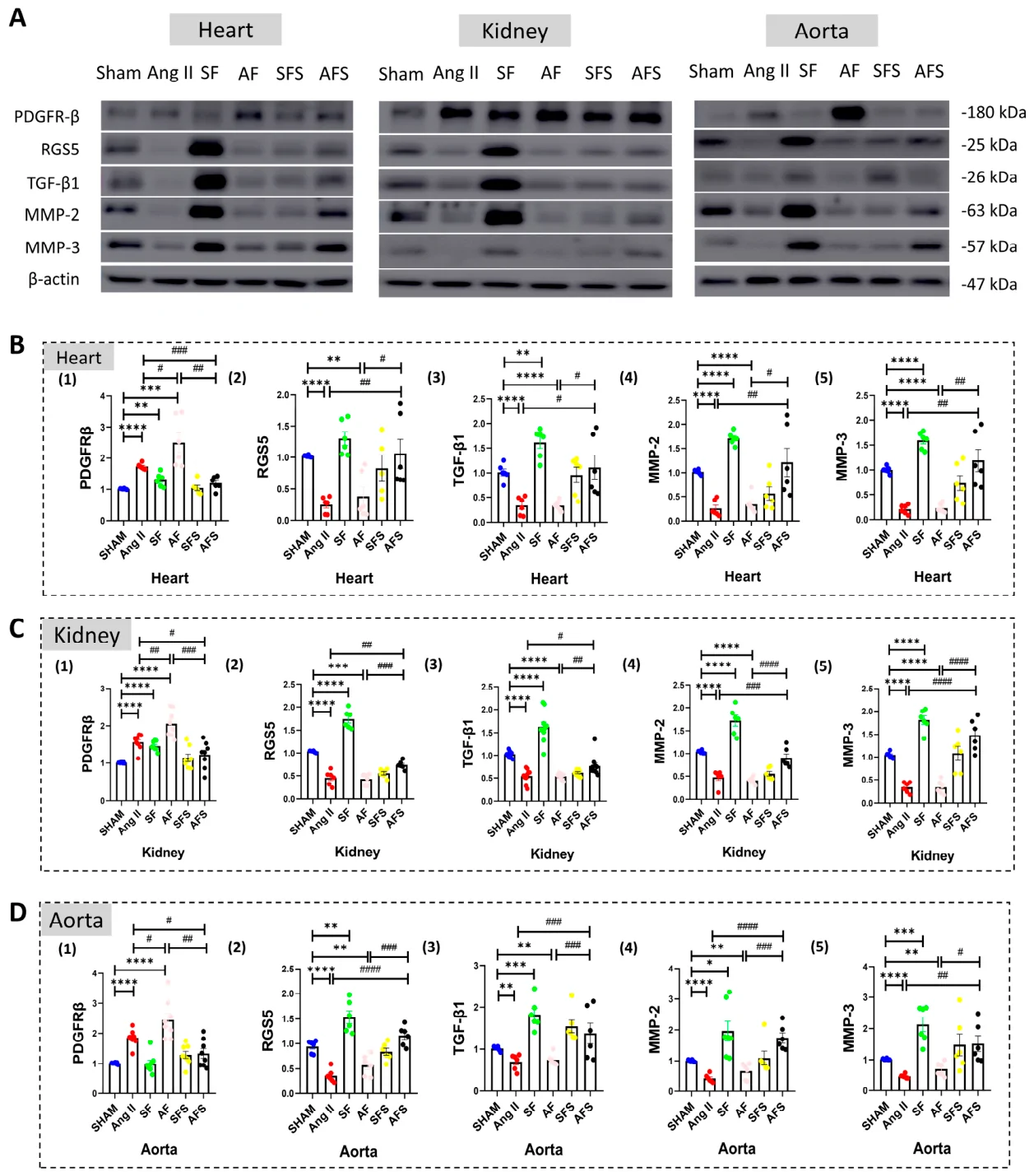
Expression of fibrosis-related proteins in the heart, kidney, and aorta determined by Western blot analysis. For panels (**B**–**D**): (**1**) PDGFRβ; (**2**) RGS5; (**3**) TGFβ1; (**4**) MMP-2; and (**5**) MMP-3. (**A**) Representative Western blot images of fibrosis-related proteins in the heart, kidney, and aorta. (**B**) Quantitative analysis of fibrosis-related protein expression in the heart. (**C**) Quantitative analysis of fibrosis-related protein expression in the kidney. (**D**) Quantitative analysis of fibrosis-related protein expression in the aorta. Values are expressed as mean ± SD. Each data point represents one individual rat. Asterisks indicate significant differences between the Sham group and the Ang II, SF, or AF groups; hash symbols indicate significant differences between the Ang II and AF or AFS groups, as well as between the AF and AFS groups. * *p* < 0.05, ** *p* < 0.01, *** *p* < 0.001, and **** *p* < 0.0001; # *p* < 0.05, ## *p* < 0.01, ### *p* < 0.001, and #### *p* < 0.0001.

Western blot analysis was performed to evaluate the expression of fibrosis-related proteins in the heart, kidney, and aorta (Figure 6). PDGFRβ expression was consistently increased in the Ang II group compared with the Sham group across all three tissues, with a further increase observed in the AF group (Figure 6B(1)–D(1)). In contrast, PDGFRβ expression was markedly reduced in the AFS group (Figure 6B(1)–D(1)). The expression of RGS5, TGFβ1, MMP-2, and MMP-3 was consistently decreased in both the Ang II and AF groups across all tissues and was partially restored in the AFS group (Figure 6B(2–5)–D(2–5)). Although TGFβ1 and MMPs are generally associated with active fibrotic remodeling, their reduced expression in the Ang II and AF groups occurred in the context of advanced fibrosis confirmed by histological analysis, suggesting a possible transition from active fibrogenesis to late-stage tissue remodeling.

### 2.5. Immunohistochemical Analysis of PDGFRβ Expression in the Heart and Kidney

**Figure 7:**
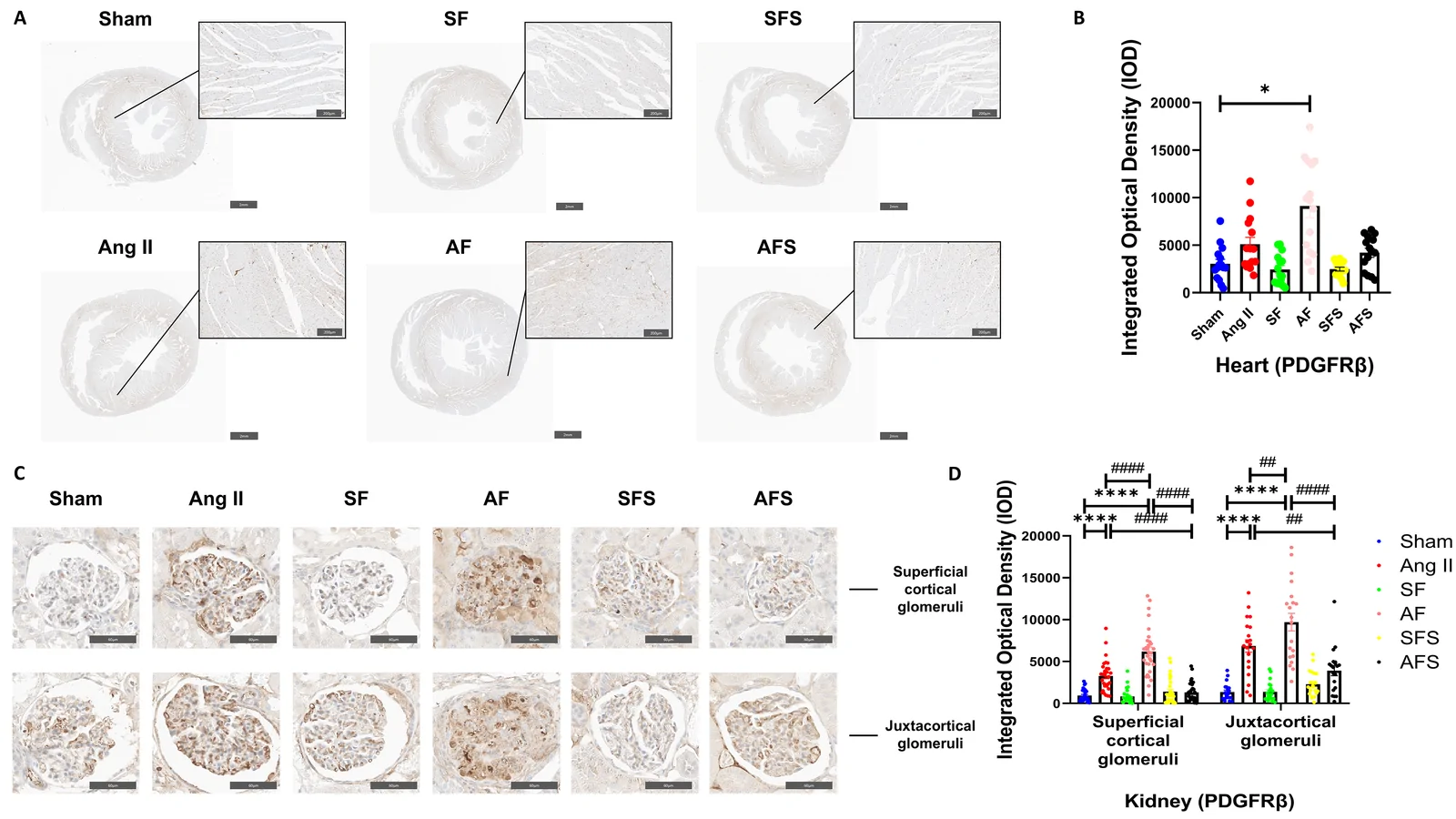
Representative PDGFRβ immunohistochemical images and quantitative analysis of PDGFRβ staining intensity in the heart and renal glomeruli. (**A**) Representative PDGFRβ immunohistochemical images of the heart. Low-magnification images are shown at ×10, and high-magnification images are shown at ×100. (**B**) Quantitative analysis of PDGFRβ staining intensity in the heart. Fifteen microscopic fields per group were obtained from tissue sections of two rats. Each data point represents one microscopic field, and values are shown as mean ± SD. Statistical comparisons were performed using nested one-way ANOVA, with the individual animal as the biological experimental unit and microscopic fields nested within-animal. Sham vs. AF, * *p* < 0.05. (**C**) Representative PDGFRβ immunohistochemical images of superficial cortical glomeruli and juxtamedullary glomeruli. Low-magnification images are shown at ×10, and high-magnification images are shown at ×400. (**D**) Quantitative analysis of PDGFRβ staining intensity in superficial cortical and juxtamedullary glomeruli. Thirty superficial cortical glomeruli and twenty juxtamedullary glomeruli per group were obtained from tissue sections of two rats. Each data point represents one glomerulus, and values are shown as mean ± SD. Separate nested one-way ANOVAs were performed for superficial cortical and juxtamedullary glomeruli, with the individual animal as the biological experimental unit and glomeruli nested within-animal. **Superficial cortical glomeruli**: Sham vs. Ang II, **** *p* < 0.0001; Sham vs. AF, **** *p* < 0.0001; Ang II vs. AF, #### *p* < 0.0001; Ang II vs. AFS, #### *p* < 0.0001; AF vs. AFS, #### *p* < 0.0001. **Juxtamedullary glomeruli**: Sham vs. Ang II, **** *p* < 0.0001; Sham vs. AF, **** *p* < 0.0001; Ang II vs. AF, ## *p* < 0.01; Ang II vs. AFS, ## *p* < 0.01; AF vs. AFS, #### *p* < 0.0001.

Given that Western blot analysis identified PDGFRβ as one of the prominently altered fibrosis- and remodeling-related proteins, immunohistochemical staining was performed to further characterize its tissue distribution in the heart and kidney. In the heart, PDGFRβ immunoreactivity was predominantly localized to the perivascular regions of intramyocardial arterioles (Figure 7A). Quantitative analysis showed significantly greater PDGFRβ staining intensity in the AF group than in the Sham group (Figure 7B). Although the Ang II group showed a higher mean staining intensity than the Sham group and the AFS group showed a lower mean value than the AF group, these differences did not reach statistical significance after nested analysis.

In the kidney, PDGFRβ immunoreactivity was predominantly localized to the glomeruli; therefore, quantitative analysis was performed separately in superficial cortical and juxtamedullary glomeruli (Figure 7C). In superficial cortical glomeruli, PDGFRβ staining intensity was significantly increased in both the Ang II and AF groups compared with the Sham group, with a further increase in the AF group compared with the Ang II group. PDGFRβ staining intensity in the AFS group was significantly lower than that in both the Ang II and AF groups (Figure 7D). A similar pattern was observed in juxtamedullary glomeruli: staining intensity was significantly increased in the Ang II and AF groups compared with the Sham group, and was further increased in the AF group compared with the Ang II group. The AFS group showed significantly lower staining intensity than both the Ang II group and AF group (Figure 7D). Detailed cardiac and renal PDGFRβ staining intensity data are provided in Supplementary Table S7.

### 2.6. Expression of MR, Ion Transport, and Inflammatory Signaling-Related Proteins

**Figure 8:**
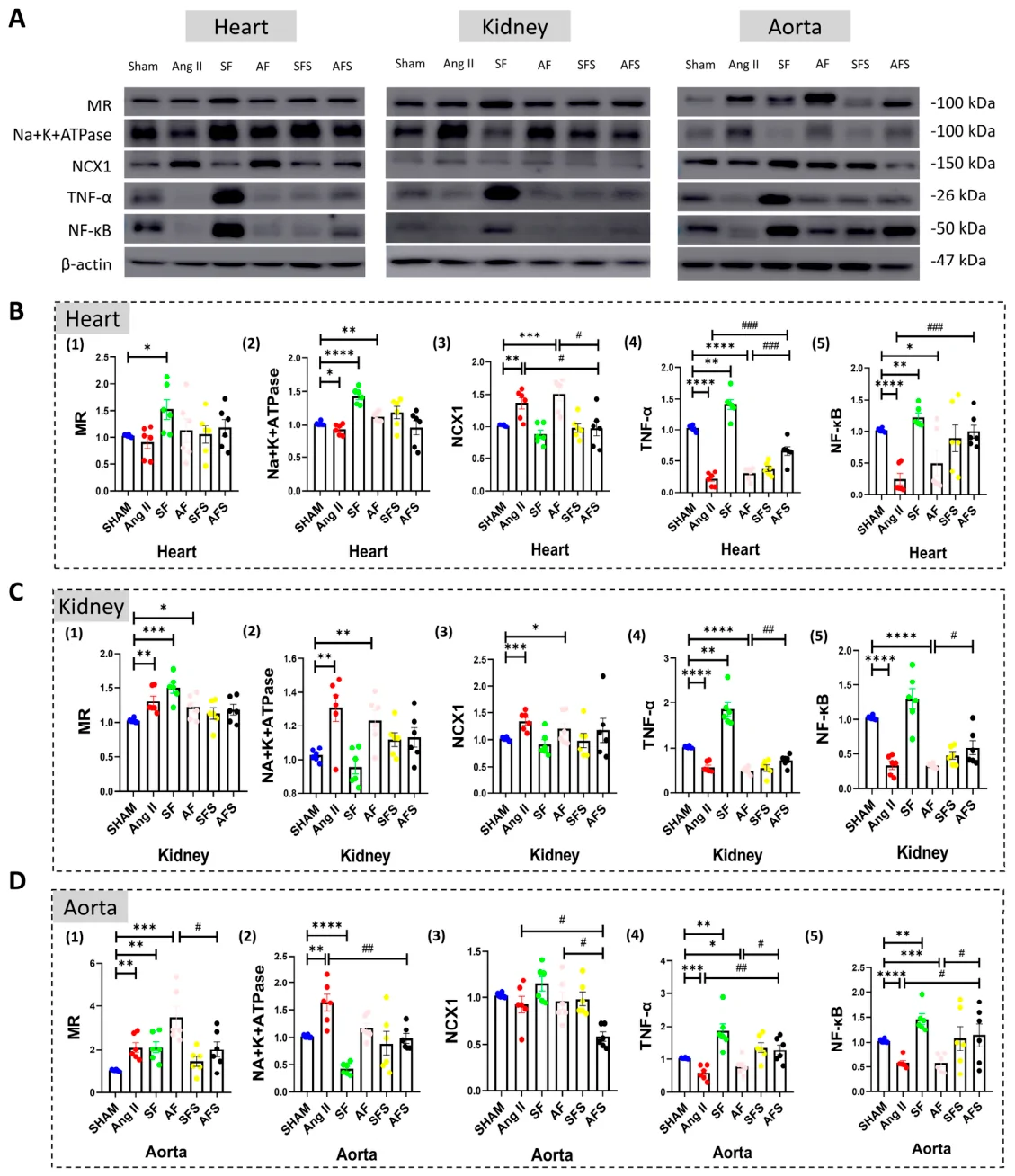
Representative Western blot images and quantitative analysis of mineralocorticoid receptor (MR), ion transport-related proteins, and inflammatory proteins in the heart, kidney, and aorta. For panels (**B**–**D**): (**1**) MR; (**2**) Na+/K+-ATPase; (**3**) NCX1; (**4**) TNF-α; and (**5**) NF-κB. (**A**) Representative Western blot images of MR, Na^+^/K^+^-ATPase, NCX1, TNF-α, and NF-κB in the heart, kidney, and aorta. (**B**) Quantitative analysis of protein expression in the heart. (**C**) Quantitative analysis of protein expression in the kidney. (**D**) Quantitative analysis of protein expression in the aorta. Values are expressed as mean ± SD. Each data point represents one individual rat. Asterisks indicate significant differences between the Sham group and the Ang II, SF, or AF groups; hash symbols indicate significant differences between the Ang II and AF or AFS groups, as well as between the AF and AFS groups. * *p* < 0.05, ** *p* < 0.01, *** *p* < 0.001, and **** *p* < 0.0001; # *p* < 0.05, ## *p* < 0.01 and ### *p* < 0.001.

To further investigate molecular alterations associated with tissue fibrosis, Western blot analysis was performed to evaluate the expression of MR, ion transport-related proteins, and inflammatory signaling-related proteins in the heart, kidney, and aorta (Figure 8A). MR expression was significantly increased in both the Ang II and AF groups in the kidney and aorta, whereas a significant recovery was observed only in the aorta of the AFS group (Figure 8C(1),D(1)). In the heart, Na^+^/K^+^-ATPase expression was decreased in the Ang II group but increased in the SF and AF groups (Figure 8B(2)). NCX1 expression was elevated in both the Ang II and AF groups and returned to near-Sham level in the AFS group (Figure 8B(3)). In the kidney, both Na^+^/K^+^-ATPase and NCX1 expression were increased in the Ang II and AF groups, whereas no apparent upregulation was observed in the AFS group (Figure 8C(2,3)). In the aorta, Na^+^/K^+^-ATPase expression was markedly increased in the Ang II group and was restored toward normal levels in the AFS group (Figure 8D(2)). Although no obvious increase in NCX1 expression was observed in any group, NCX1 expression in the AFS group was lower than that in both the Ang II and AF groups (Figure 8D(3)). Inflammatory signaling-related proteins, including TNF-α and NF-κB, were generally decreased in the Ang II and AF groups across all tissues and were partially restored in the AFS group (Figure 8B(4,5)–D(4,5)).

## 3. Discussion

The present study showed that, in Ang II-induced hypertension characterized by reduced PV, chronic loop diuretic treatment with furosemide did not provide additional antihypertensive benefit but was associated with further PV contraction and more severe cardiovascular and renal injury. In contrast, concomitant MR blockade with spironolactone attenuated PV contraction, lowered blood pressure, preserved vascular integrity, and ameliorated target-organ fibrosis. Although the cardiovascular and renal protective effects of MR blockade are well-established, the present study extends these observations by integrating direct longitudinal assessment of PV with simultaneous evaluation of blood pressure and structural, endothelial, and molecular remodeling during chronic loop diuretic treatment under sustained Ang II activation. Notably, further PV contraction in furosemide-treated hypertensive rats occurred in parallel with aggravated target-organ injury despite the absence of additional blood pressure reduction, whereas MR blockade was accompanied by attenuation of both PV contraction and tissue injury. These parallel changes support a potential relationship between circulating volume homeostasis and target-organ remodeling in this setting.

Loop diuretics, in principle, lower blood pressure primarily through sodium and water excretion, making them highly effective in conditions characterized by volume expansion [17]. However, increasing evidence indicates that hypertension is not always accompanied by expanded PV [7]. Patients with high-renin hypertension and experimental models driven by excessive Ang II activity often exhibit relative contraction of effective circulating PV despite sustained hypertension [18,19]. The mechanisms underlying PV contraction during chronic Ang II exposure are likely complex. Ang II has well-established sodium- and water-retaining actions, including direct stimulation of renal tubular sodium reabsorption, stimulation of aldosterone secretion, and promotion of vasopressin release [20,21]. These responses would be expected to counteract volume depletion. However, chronic Ang II-induced hypertension also markedly alters renal and systemic hemodynamics, and the resulting elevation in arterial pressure modifies renal sodium and water excretion through pressure-dependent mechanisms [22,23]. Thus, the final PV reflects the net balance among renal sodium handling, neurohumoral regulation, arterial pressure, and vascular fluid distribution rather than the action of a single pathway. In the present study, Ang II-treated rats showed increased urine output and water intake together with body-weight loss and reduced PV, suggesting substantial disruption of fluid homeostasis. Nevertheless, because urinary sodium balance, circulating aldosterone and vasopressin, and renal hemodynamics were not directly measured, the relative contributions of these mechanisms to PV contraction cannot be determined from the present data. Under these conditions, vascular resistance rather than circulating volume becomes the major determinant of arterial pressure [24]. Consistent with this concept, furosemide did not reduce blood pressure in Ang II-infused rats despite producing marked PV contraction. Instead, excessive volume depletion was accompanied by aggravated myocardial degeneration, renal injury, and tissue fibrosis, suggesting that further reduction in PV may become detrimental when effective circulating volume is already compromised. The supplementary NHANES analysis provided additional clinical context, showing that concomitant MR blockade was associated with lower SBP after multivariable adjustment, whereas the association with DBP did not reach statistical significance. The more pronounced association with SBP than with DBP is broadly consistent with previous clinical evidence showing a greater absolute reduction in SBP than in DBP with MR antagonist treatment [25]. Nevertheless, given the observational nature of the NHANES analysis, these findings should be interpreted as supportive clinical context rather than evidence of a treatment effect.

The mechanisms underlying these findings are likely multifactorial. From a physiological perspective, excessive PV contraction could potentially compromise tissue perfusion and induce compensatory neurohumoral responses, including activation of the RAAS and sympathetic nervous system, which may contribute to vasoconstriction, endothelial dysfunction, and fibrotic remodeling [19,26]. Notably, organ injury was substantially more severe in the AF group despite blood pressure levels comparable to those of the Ang II group. This observation suggests that PV status itself may influence target-organ injury independent of blood pressure and highlights the importance of maintaining adequate effective circulating volume during chronic RAAS activation [7,18]. However, renal blood flow, tissue perfusion, circulating RAAS activity, and sympathetic nerve activity were not directly measured in the present study. Therefore, these mechanisms should be regarded as plausible interpretations rather than direct experimental findings.

An important finding of the present study is that MR blockade largely prevented the detrimental consequences of loop diuretic-induced PV contraction. In addition to lowering BP, spironolactone markedly preserved PV and attenuated pathological remodeling in the heart, kidney, and aorta. These protective effects are consistent with the well-recognized actions of MR antagonists beyond BP reduction, including improvement of endothelial function and inhibition of tissue fibrosis [27,28]. Our data further suggest that preservation of effective circulating PV may represent an additional mechanism contributing to the cardiovascular and renal protection afforded by MR blockade.

In the present study, our Ang II-induced hypertensive rat model exhibited marked cardiovascular and renal structural injury, including narrowing and focal occlusion of intramyocardial vessels in the heart and glomerular damage in the kidney. These pathological changes were further aggravated by furosemide treatment in the AF group and were accompanied by reduced eNOS expression and enhanced PDGFRβ staining. Taken together, these findings indicate that the vascular abnormalities observed in this experimental model involve extensive vascular remodeling rather than isolated endothelial dysfunction. In parallel, MR blockade partially restored the expression of RGS5 and fibrosis-related proteins, including MMP-2, MMP-3, and TGF-β1, suggesting partial normalization of vascular and extracellular matrix homeostasis. Although these molecules are commonly involved in active tissue injury and fibrogenesis, their expression is highly dependent on the tissue context and stage of fibrosis [29,30]. In particular, MMPs may exhibit either increased or decreased expression during different phases of fibrotic remodeling, reflecting alterations in extracellular matrix turnover and cellular activity [31,32]. Therefore, the reduced expression observed in the Ang II and AF groups despite extensive collagen deposition may reflect advanced chronic remodeling rather than the absence of ongoing fibrosis. In addition, MR blockade markedly restored the expression of these proteins in Ang II- and AF-treated rats, in which severe tissue fibrosis had already been confirmed by histological analysis. Collectively, these findings raise the possibility of a bidirectional relationship between vascular remodeling and PV contraction. Progressive vascular remodeling, luminal narrowing, and loss of vascular compliance may impair the capacity of the vascular system to maintain effective circulating volume [33]. Conversely, excessive PV contraction may compromise tissue perfusion and enhance compensatory neurohumoral activation, thereby further promoting endothelial dysfunction and fibrotic remodeling [30,34]. Thus, vascular injury, PV contraction, and tissue fibrosis may interact to establish a self-perpetuating cycle during chronic Ang II stimulation. Importantly, however, the present experimental design cannot determine the direction of causality within this relationship. Therefore, the parallel improvement in PV, vascular integrity, and tissue injury observed following MR blockade should be interpreted as evidence of an association among these processes rather than proof that PV preservation directly mediates the protective effects of MR blockade.

Alterations in Na^+^/K^+^-ATPase and NCX1 further suggest that ionic remodeling contributes to hypertensive target organ injury in a tissue-specific manner [35]. In the heart, Ang II predominantly increased NCX1 expression, whereas furosemide treatment was associated with enhanced Na^+^/K^+^-ATPase expression, indicating distinct mechanisms of calcium and sodium handling during cardiac remodeling. In contrast, both Na^+^/K^+^-ATPase and NCX1 were upregulated in the kidney following Ang II infusion, suggesting coordinated disturbances in sodium and calcium homeostasis during renal injury. Although changes in the aorta were less pronounced, MR blockade consistently restored the expression of these ion transporters toward control levels. These findings suggest that the protective effects of MR blockade may involve, at least in part, the normalization of tissue-specific ionic homeostasis, thereby limiting cellular dysfunction and fibrotic remodeling [23,30].

In addition, TNF-α and NF-κB exhibited similar expression patterns across the heart, kidney, and aorta, with reduced expression in the Ang II and AF groups and partial restoration following MR blockade. Although activation of TNF-α/NF-κB signaling has been widely implicated in Ang II-induced hypertension and tissue injury, inflammatory signaling is highly dynamic and may vary according to the duration and severity of tissue injury [36,37]. Histological analysis in the present study demonstrated pronounced myocardial degeneration, glomerulosclerosis, tubular injury, and extensive fibrosis in the Ang II and particularly the AF groups. Therefore, advanced tissue remodeling and associated alterations in cellular composition may have contributed to the reduced abundance of these proteins in whole-tissue lysates [29,30]. However, because apoptosis, cell viability, and inflammatory cell populations were not directly assessed, this possibility remains speculative. Alternative mechanisms should also be considered. NF-κB signaling is subject to extensive negative-feedback and post-translational regulation, and changes in total NF-κB protein abundance do not necessarily parallel its transcriptional activity, which is also determined by phosphorylation, degradation, and nuclear translocation. Regulation of TNF-α synthesis, processing, and turnover may similarly influence its tissue protein abundance. Thus, the reduced TNF-α and NF-κB levels observed in the Ang II and AF groups may reflect a combination of stage-dependent remodeling, altered tissue cellular composition, and adaptive regulation of inflammatory signaling rather than a simple reduction in inflammatory activity. Interestingly, fibrosis- and inflammation-related proteins were consistently altered in multiple tissues of the SF group despite the absence of apparent histopathological abnormalities. These findings suggest that furosemide treatment alone may elicit early molecular responses before overt structural injury becomes detectable. From a clinical perspective, this observation raises the possibility that loop diuretics may induce subclinical molecular or neurohumoral adaptations even in the absence of hypertension or apparent tissue injury. Previous studies in healthy normotensive subjects have similarly demonstrated activation of the renin–angiotensin–aldosterone and sympathetic nervous systems following furosemide administration, even without a substantial reduction in blood pressure [38,39]. However, because the SF group showed neither significant PV contraction nor detectable histopathological injury, these molecular alterations should be interpreted as potential early adaptive responses rather than evidence of direct tissue damage. Further studies are required to determine whether such changes persist or progress during prolonged loop diuretic exposure.

The present findings may have important clinical implications. Current antihypertensive therapy is largely guided by BP, whereas PV status is rarely considered when selecting diuretic treatment [3,34]. Our results suggest that patients with hypertension characterized by excessive RAAS activation and relative PV contraction may derive limited benefit from further aggressive diuresis. Instead, therapeutic strategies capable of suppressing pathological RAAS signaling while preserving effective circulating PV may provide greater protection against target-organ injury. These findings support the concept that individualized assessment of PV status may help optimize antihypertensive therapy in selected patients.

Several limitations should be acknowledged. First, the present study was conducted exclusively in male rats using an Ang II-induced hypertension model, which may limit the generalizability of the findings to female animals and other forms of hypertension. In addition, the Ang II infusion rate used in this study (800 ng/kg/min) represents a relatively high pressor dose and was intended to induce pronounced and sustained Ang II/RAAS activation together with target-organ remodeling. This dose is likely to produce circulating Ang II exposure above physiological levels; however, plasma Ang II concentrations were not directly measured, and the magnitude of this elevation therefore cannot be quantified. Accordingly, the present model should be regarded as representing pronounced Ang II/RAAS activation rather than directly reproducing the circulating Ang II levels typically observed in human hypertension. Second, although PV was directly quantified using the ICG indicator-dilution technique, several complementary physiological measurements were not obtained. Circulating RAAS activity, red cell volume, and total blood volume were not directly measured, and renal AQP2 expression and circulating AVP levels were not assessed. These measurements could provide additional information regarding neurohumoral regulation, renal water handling, and changes in the overall circulating blood compartment. In addition, although early individual back-extrapolation was used to account for ICG disappearance, hepatic function and perfusion were not independently assessed; therefore, a potential influence of between-group differences in hepatic ICG clearance on PV estimation cannot be completely excluded. Third, although the parallel changes in PV and target-organ injury observed in this study support a close relationship between these processes, the present experimental design does not establish causality or determine its direction. PV contraction may compromise tissue perfusion and contribute to subsequent organ injury, whereas progressive vascular remodeling, luminal narrowing, and reduced vascular compliance may in turn impair the ability of the circulation to maintain effective circulating volume. These processes may therefore interact bidirectionally and potentially form a self-reinforcing cycle during sustained Ang II activation. Controlled restoration or manipulation of PV could provide additional mechanistic insight; however, such interventions would also alter cardiac preload, arterial pressure, renal hemodynamics, and neurohumoral responses. Future studies combining controlled manipulation of circulating volume with direct assessment of tissue perfusion and microvascular function, while accounting for these hemodynamic and neurohumoral changes, will be required to clarify the direction and mechanisms of this relationship. Finally, quantitative histological and immunohistochemical analyses were based on tissue sections from two animals per group. Although nested analyses were used to account for within-animal clustering and avoid pseudo replication, the limited number of biological replicates remains an important limitation. Sampling multiple microscopic fields or glomeruli improves the characterization of within-tissue spatial heterogeneity but does not substitute for independent biological replication; therefore, these quantitative histological and immunohistochemical findings should be interpreted with appropriate caution and confirmed in future studies using larger numbers of animals.

## 4. Materials and Methods

### 4.1. Animals

A total of 36 male Sprague–Dawley (SD) rats, approximately 12 weeks of age, were obtained from the Bio Medical Hub, College of Medicine, Seoul National University. During the experimental period, the rats were housed in plastic cages (1 animal per cage) under controlled environmental conditions (temperature: 21–23 °C; relative humidity: 50–60%) with a 12 h light/12 h dark cycle.

### 4.2. PV Measurement

PV was measured in all rats on the first experimental day (Day 1) and repeated immediately before terminal tissue collection under anesthesia on the final experimental day (Day 35) (Figure 1A). PV was determined using the ICG (Tokyo Chemical Industry Co., Ltd., Tokyo, Japan; Cat. No. I0535) indicator dilution technique as previously described by other experimental teams [40,41]. During each PV measurement procedure, an indwelling venous catheter was maintained for both ICG administration and serial blood sampling. After collection of a baseline blood sample, ICG (0.1 mg/mL) was administered as an intravenous bolus via the jugular venous catheter at a dose of 0.25 mg/kg body weight, followed immediately by a saline flush to ensure complete delivery of the dye.

Sequential blood samples were collected through the same jugular venous catheter beginning 2 min after ICG injection and subsequently at 30 s intervals for a total of six samples (2.0, 2.5, 3.0, 3.5, 4.0, and 4.5 min after injection). Before each blood collection, the catheter dead-space volume was discarded to avoid contamination with residual ICG solution. Plasma was separated by centrifugation at 3000× *g* for 15 min at 4 °C. Plasma absorbance was measured at 805 nm using a SPARK microplate reader (Ref. No. 30086376; Tecan Austria GmbH, Grödig, Austria), and plasma ICG concentrations were determined from a standard calibration curve prepared using known ICG concentrations.

The natural logarithm of plasma ICG concentration was plotted against time, and linear regression analysis was performed using Microsoft Excel 2019 (Microsoft Corp., Redmond, WA, USA). The regression line was back-extrapolated to estimate the theoretical plasma ICG concentration immediately after complete intravascular mixing (C_0_). PV was then calculated according to the indicator dilution principle as follows:

PV = (Injected ICG dose)/C_0_

where the injected ICG dose represents the total amount of ICG administered and C_0_ is the extrapolated plasma ICG concentration at time zero. Although the complete plasma concentration–time profile of ICG may exhibit biphasic or multiexponential kinetics, the early disappearance phase can be distinguished from the slower later phase. Following intravenous administration, ICG is rapidly bound to plasma proteins and selectively extracted from the circulation by hepatocytes, followed by excretion of unchanged ICG into the bile. Consequently, later concentration–time data may increasingly reflect slower hepatobiliary elimination kinetics. In contrast, during the early post-injection period, after adequate intravascular mixing, plasma ICG disappearance can be reliably approximated by a monoexponential decay. Previous methodological studies have demonstrated that monoexponentiality is particularly reliable within approximately the first 5 min after injection and that inclusion of later time points may influence back-extrapolation of the initial concentration [41,42,43]. Therefore, consistent with established ICG plasma-volume measurement methods, only concentrations obtained from 2.0 to 4.5 min were fitted using log-linear regression and back-extrapolated to estimate C_0_.

### 4.3. Experimental Protocol

One week after the initial PV measurement, rats were randomly assigned to six experimental groups (n = 6 per group): Sham (S), Ang II (A), Sham + furosemide (SF), Ang II + furosemide (AF), Sham + furosemide + spironolactone (SFS), and Ang II + furosemide + spironolactone (AFS). Ang II (Cat. No. 05-23-0101; Calbiochem, Merck KGaA, Darmstadt, Germany) was continuously infused at 800 ng/kg/min using an osmotic pump (2004W, 200 μL, 4-week duration; RWD Life Science Co., Ltd., Shenzhen, Guangdong, China). This pressor dose was selected based on established Ang II infusion models employing comparable doses to induce sustained hypertension and target-organ remodeling [44,45,46]. One week after group allocation (Week 3 of the experimental protocol), furosemide (20 mg/tablet; Shanghai Zhaohui Pharmaceutical Co., Ltd., Shanghai, China) was administered by oral gavage once daily at 50 mg/kg/day to the SF, AF, SFS, and AFS groups, whereas spironolactone (20 mg/tablet; Zhejiang Yatai Pharmaceutical Co., Ltd., Shaoxing, Zhejiang, China) was additionally administered once daily at 100 mg/kg/day to the SFS and AFS groups. The furosemide dose (50 mg/kg/day) was selected within the range of pharmacologically active doses previously used for chronic loop diuretic treatment in rats, in which oral or dietary doses of approximately 30–50 mg/kg/day have been shown to produce sustained diuretic and renal effects [47,48,49]. Spironolactone (100 mg/kg/day) was selected based on previous rat studies demonstrating effective MR antagonism and attenuation of cardiovascular or renal remodeling at this dose [49,50,51]. These doses were intended to provide sustained loop diuretic exposure and robust MR blockade, respectively, rather than to model human-equivalent clinical doses. Following group allocation, systolic and diastolic BP were measured twice weekly using the CODA non-invasive tail-cuff BP system (Model CODA; Kent Scientific Corporation, Torrington, CT, USA). Rats were also housed in metabolic cages twice weekly for the measurement of water intake, urine output, and body weight. Five weeks after the initial PV measurement, PV was reassessed. The animals were then deeply anesthetized and euthanized, after which the heart, kidneys, and aorta were collected (Figure 1A).

### 4.4. NHANES Cohort and Statistical Analysis

Data from the National Health and Nutrition Examination Survey (NHANES) 1999–2018 cycles were used to provide complementary clinical context for the experimental findings. Among 101,316 participants, individuals without a diagnosis of hypertension were excluded. Hypertensive participants receiving loop diuretics for at least 30 days were subsequently identified and classified according to concomitant MR blockade, resulting in 1473 eligible participants (loop diuretic only, n = 1337; loop diuretic plus MR blockade, n = 136). The detailed participant selection process is presented in Supplementary Figure S2.

SBP and DBP were initially compared between the two groups using *t*-test. Multivariable linear regression analyses were subsequently performed using the loop diuretic-only group as the reference. Model 1 was unadjusted; Model 2 was adjusted for age, gender, and BMI; and Model 3 was additionally adjusted for heart failure, diabetes mellitus, eGFR, RBC, and WBC. Regression coefficients (B) with 95% confidence intervals (CIs) and corresponding *p* values were reported.

### 4.5. Hematoxylin and Eosin (H&E) and Masson’s Trichrome Staining

Heart, kidney, and aortic tissue samples were fixed in 4% paraformaldehyde, embedded in paraffin, and cut into 4 μm thick sections. The sections were stained with H&E and Masson’s trichrome. H&E-stained sections were imaged using an Olympus IX53 microscope (Olympus Corp., Tokyo, Japan) to evaluate morphological changes in each group. Masson’s trichrome-stained sections were scanned using a Leica SCN400F slide scanner (Leica Microsystems, Wetzlar, Germany). The fibrotic area was quantified using Aperio ImageScope (version 12.4.6, Leica Biosystems, Buffalo Grove, IL, USA) and ImageJ (version 1.52p, National Institutes of Health, Bethesda, MD, USA), and the results were expressed as a percentage of the total analyzed area.

To further characterize the distribution of fibrosis severity across microscopic fields, an exploratory threshold-based analysis was performed. For each tissue, the mean fibrotic area of all microscopic fields from the Sham group was defined as the first threshold (T1). For the heart, progressively higher thresholds were defined as 1.5-, 2.0-, and 3.0-fold T1 (T2–T4, respectively), corresponding to 3.18%, 4.77%, 6.36%, and 9.54% fibrotic area. For the renal cortex, T2–T4 were defined as 1.2-, 1.5-, and 1.8-fold T1, corresponding to 7.54%, 9.05%, 11.31%, and 13.57%, respectively. The same fold increments were applied to the renal medulla, yielding thresholds of 8.97%, 10.76%, 13.46%, and 16.15%, respectively. For each experimental group, the proportion of microscopic fields with a fibrotic area exceeding each threshold was calculated. This threshold-based analysis was used descriptively to visualize the distribution of fibrosis severity and was not subjected to inferential statistical testing.

### 4.6. Western Blotting Analysis

Total protein was extracted from heart, kidney, and aortic tissues using 1× RIPA buffer (CellNest, Hanam-si, Republic of Korea; Cat. No. CNR001-0100) containing protease and phosphatase inhibitor cocktails (pH 7.4). Protein concentrations were determined using the bicinchoninic acid (BCA) assay, and protein samples were mixed with 5× loading buffer. The samples were denatured at 95 °C for 10 min, separated by SDS-PAGE, and transferred onto polyvinylidene difluoride (PVDF) membranes using transfer buffer containing 25 mM Tris, 192 mM glycine, 0.01% SDS, and 20% methanol. The membranes were blocked for 1 h at room temperature with Tris-buffered saline containing 0.1% Tween-20 (TBST) supplemented with 5% bovine serum albumin (BSA), followed by overnight incubation with primary antibodies at 4 °C. Primary antibodies included PDGFRβ (1:1000, sc-374573, Santa Cruz Biotechnology, Dallas, TX, USA), RGS5 (1:1000, sc-390245, Santa Cruz Biotechnology, Dallas, TX, USA), TGFβ1 (1:1000, sc-130348, Santa Cruz Biotechnology, Dallas, TX, USA), MMP-2 (1:1000, sc-53630, Santa Cruz Biotechnology, Dallas, TX, USA), MMP-3 (1:1000, sc-271230, Santa Cruz Biotechnology, Dallas, TX, USA), MR (1:1000, sc-53000, Santa Cruz Biotechnology, Dallas, TX, USA), Na^+^/K^+^-ATPase (1:1000, sc-21712, Santa Cruz Biotechnology, Dallas, TX, USA), NCX1 (1:1000, ab240203, Abcam, Cambridge, UK), TNF-α (1:1000, sc-52746, Santa Cruz Biotechnology, Dallas, TX, USA), NF-κB (1:1000, sc-514451, Santa Cruz Biotechnology, Dallas, TX, USA), and β-actin (1:1000, sc-47778, Santa Cruz Biotechnology, Dallas, TX, USA). After washing, the membranes were incubated with HRP-conjugated secondary antibodies, including anti-mouse IgG, HRP-linked antibody (#7076; Cell Signaling Technology, Danvers, MA, USA) and anti-rabbit IgG, HRP-linked antibody (#7074; Cell Signaling Technology, Danvers, MA, USA). Protein bands were detected using an enhanced chemiluminescence (ECL) detection kit (Amersham Biosciences, Little Chalfont, UK). Band intensities were quantified using ImageJ and normalized to the corresponding β-actin signal.

### 4.7. Immunohistochemical Analysis

Paraffin-embedded heart and kidney tissue sections were deparaffinized, rehydrated, and subjected to antigen retrieval in 10 mM sodium citrate buffer (pH 6.0) at 95 °C for 20 min. After blocking with normal goat serum for 1 h at room temperature, the sections were incubated overnight at 4 °C with primary antibodies against eNOS (1:300, sc-376751, Santa Cruz Biotechnology, Dallas, TX, USA) and PDGFRβ (1:1000, sc-374573, Santa Cruz Biotechnology, Dallas, TX, USA). The sections were then incubated with the appropriate HRP-conjugated secondary antibodies for 30 min at room temperature. Immunoreactivity was visualized using a 3,3′-diaminobenzidine (DAB) substrate kit (Sigma-Aldrich, St. Louis, MO, USA) according to the manufacturer’s instructions. After mounting, the sections were scanned using a Leica SCN400F slide scanner (Leica Microsystems, Wetzlar, Germany). Immunohistochemical staining was quantitatively analyzed using Aperio ImageScope (version 12.4.6, Leica Biosystems, Buffalo Grove, IL, USA) and ImageJ (version 1.52p, National Institutes of Health, Bethesda, MD, USA).

Because eNOS and PDGFRβ are predominantly expressed in vascular endothelial cells and perivascular cells, respectively, representative images were captured from ×10 and ×100 fields containing abundant intramyocardial arterioles in heart sections and from ×400 glomerular fields in kidney sections. Immunoreactivity was quantified as the integrated optical density (IOD).

For quantitative histological and immunohistochemical analyses, tissue sections obtained from two rats in each experimental group were evaluated. Multiple microscopic fields or glomeruli were selected from each animal to assess regional and spatial heterogeneity within the tissues. The number of microscopic fields or glomeruli analyzed varied according to the tissue and anatomical region and is specified in the corresponding figure legends. In the graphical presentation, each data point represents an individual microscopic field or glomerulus.

To further evaluate glomerular structural alterations, the diameters of individual glomeruli and their corresponding Bowman’s capsules were measured using Aperio ImageScope (version 12.4.6, Leica Biosystems, Buffalo Grove, IL, USA). Glomerular and Bowman’s capsule volumes were estimated by assuming an approximately spherical geometry and applying the standard sphere-volume formula (V = 4/3πr^3^), where (r) represents one-half of the measured diameter. The glomerular-to-Bowman’s capsule volume ratio was subsequently calculated as glomerular volume divided by Bowman’s capsule volume and expressed as a percentage. Superficial cortical and juxtamedullary glomeruli were analyzed separately. The numbers of glomeruli analyzed in each anatomical region are provided in the corresponding figure legend.

### 4.8. Statistical Analysis

Statistical analyses were performed using GraphPad Prism 8 (GraphPad Software, San Diego, CA, USA). Data are presented as the mean ± standard deviation (SD). Longitudinal measurements, including systolic blood pressure, diastolic blood pressure, urine output, water intake, and body weight, were analyzed using two-way repeated-measures ANOVA, with treatment group as the between-subject factor and time as the within-subject factor, followed by appropriate multiple-comparison testing. Endpoint animal-level comparisons among multiple independent groups were performed using one-way ANOVA followed by Tukey’s multiple-comparisons test, and the unpaired Student’s *t*-test was used for comparisons between two independent groups, where appropriate. For quantitative histological and immunohistochemical analyses in which multiple microscopic fields or glomeruli were obtained from each animal, nested one-way ANOVA was used, with the individual animal as the biological experimental unit and microscopic fields or glomeruli nested within-animal. A two-tailed *p* < 0.05 was considered statistically significant.

## 5. Conclusions

Loop diuretic treatment aggravated PV contraction and target-organ injury in Ang II-induced hypertension without providing additional BP reduction, whereas MR blockade was associated with attenuation of PV contraction and reduced cardiovascular and renal damage. These findings suggest that preservation of effective circulating PV may contribute to, rather than necessarily mediate, the protective effects observed with MR blockade. These experimental findings further raise the possibility that PV status may be relevant when evaluating diuretic therapy in selected forms of hypertension characterized by marked RAAS activation. However, because this conclusion is derived primarily from an animal model and PV-guided treatment was not evaluated clinically, prospective clinical studies are required to determine whether assessment of PV status can improve the selection or personalization of diuretic therapy.

## Data Availability

The raw data supporting the findings of this study are available from the corresponding author upon reasonable request.

## Supplementary Materials

The following supporting information can be downloaded at https://www.mdpi.com/article/10.3390/ijms27188251/s1.

## Abbreviations

The following abbreviations are used in this manuscript:

Ang II	Angiotensin II
BP	Blood pressure
DBP	Diastolic blood pressure
H&E	Hematoxylin and eosin
ICG	Indocyanine green
MR	Mineralocorticoid receptor
PV	Plasma volume
RAAS	Renin–angiotensin–aldosterone system
SBP	Systolic blood pressure
