# Supplementary material for: Mineralocorticoid Receptor Blockade Mitigates Loop Diuretic-Exacerbated Plasma Volume Contraction and Target-Organ Fibrosis in Angiotensin II-Induced Hypertension
Source: Int J Mol Sci. 2026 Sep 16;27(18):8251. doi: 10.3390/ijms27188251 (PMC13607756; doi:10.3390/ijms27188251)
Supplement: Supplementary file 1 [file ijms-27-08251-s001.zip › Supplementary Information.pdf]

Supplementary Information

Supplementary Figure 1

Comparison of glomerular and Bowman's capsule volumes in superficial cortical and juxtacortical glomeruli

Supplementary Figure 2

Patient selection and classification process for the NHANES cohort

Supplementary Table 1

Detailed Blood Pressure Comparison Results from the NHANES Cohort

Supplementary Table 2

Detailed plasma volume measurements in each group on Day 1 and Day 35, and the change in PV (Day 35 − Day 1)

Supplementary Table 3

Detailed hierarchical statistical analyses of histological and immunohistochemical measurements.

Supplementary Table 4

Detailed fibrotic area measurements in the heart, renal cortex and renal medulla of each group of rats

Supplementary Table 5

Detailed eNOS staining intensity data for the heart, superficial cortical and juxtacortical

glomeruli in each group of rats

Supplementary Table 6

Detailed measurements of glomerular volume, Bowman's capsule volume, and the glomerular-to-Bowman's capsule volume ratio in superficial cortical and juxtacortical glomeruli of each group of rats

Supplementary Table 7

Detailed PDGFRβ staining intensity data for the heart, superficial cortical and juxtacortical glomeruli in each group of rats
